# Example before Precept

**Published:** 1890-07-26

**Authors:** 


### Passing Topics.

### EXAMPLE BEFORE PRECEPT.

It will be remembered that a certain gentleman who, whatever his other disappointments, had no reason at one time to complain of a want of attention, delivered himself into the public ear to the effect that some people have money and some have brains. We believe that the conclusion he desired to establish was that the people who have money are the natural and legitimate quarry of those who have brains—a postulate doubtless more justified by its truth than its morality. Without adopting the sentiment in all its native force, or perhaps it might be better to say, without attaching to it the significance it acquired in the mouth of its author, we may describe the reflection as one appropriate to the sentiments of the authorities of impecunious charities, more particularly upon the occasion of the perennial appeal.

But though it is true enough that the burden of being rests heavily upon many valuable institutions, which are kept alive only by the wits of their managers, it is equally true that others are the recipients of a practical sympathy, which appears to place them beyond the reach of financial embarrassment. We recently took occasion to congratulate one provincial hospital upon the possession of two good and generous friends, but we have been reminded of a still more notable instance of a ready and redoubtable co-operation upon the part of those who, although most intimately connected with the charity, are sometimes backward in giving proof of the vitality of their interest—we mean the members of the managing committee.

To all who are disposed to agree that the members of a committee should be prepared to do themselves, according to their means, what they are quick to ask of other people, we commend the report of the Liverpool Royal Infirmary, which furnishes the best illustration within our knowledge of the application of this unassailable principle. The Royal Infirmary, as is well known, has been recently rebuilt, and at a cost, we believe, of about £100,000. Of this sum, before a brick was laid, the twenty-six members of the committee subscribed—we are not told what further sum, if any, they collected—nearly £25,000. This total was made up of sums ranging from £7,500 to £50, each contribution, doubtless, reflecting in a measure the means of the particular donor, and being as honourable in the case of the smallest as of the largest benefaction.

Of course it would not be in the power of every or many committees to provide funds in this munificent fashion, and it is equally obvious that it would be both impolitic and unfair to exclude all but wealthy men from a share in the management. Time and personal attention and interest may be sometimes almost as valuable as more material gifts, but then these are not always forthcoming, even when the latter are wanting, and for the reason, perhaps, that just as subscribers to charities constitute but a small portion of the well-to-do population, and appear and reappear in the lists of many institutions, so members of committees, treasurers, and others often associate their names with a host of undertakings, and dissipate the energies which gain their chief power by concentration.

Perhaps the London Homœopathic Hospital, standing singly as it does, has just furnished a good case in point. We are not concerned at this moment with the relative claims to public confidence of homœopathy and legitimate medicine, but some allopathic institutions might well imitate the solicitude of the homœopathists in regard to their hospital. In the lists of donations towards rebuilding, published a few days ago, we observe that the treasurer not only subscribes £2,000 to the fund, but gives £1,000 in the name of his wife, and brings in a friend who contributes £10,000, and other substantial helpers. The aspiration of many a hospital worker will be — would that all treasurers were like the treasurer of the London Homœopathic Hospital, and all committees like the committee of the Liverpool Royal Infirmary! The force lent to the appeals of these institutions might possibly be vouchsafed to those of others, did their managers only deny themselves the impotent luxury of dallying with many charities instead of identifying themselves heart and soul with a few.

